# Paper-Based Sensors: Fantasy or Reality?

**DOI:** 10.3390/nano15020089

**Published:** 2025-01-08

**Authors:** Ghenadii Korotcenkov

**Affiliations:** Department of Physics and Engineering, Moldova State University, MD-2009 Chisinau, Moldova; ghkoro@yahoo.com; Tel.: +373-60642109

**Keywords:** paper properties, humidity sensors, electrochemical sensors, gas sensors, biosensors, pressure sensors, analytical devices, limitations

## Abstract

This article analyzes the prospects for the appearance of paper-based sensors on the sensor market. It is concluded that paper-based sensors are not a fantasy but a reality. It is shown that paper has properties that make it possible to develop a wide variety of paper-based sensors, such as SERS, colorimetric, fluorescent, conductometric, capacitive, fiber-optic, electrochemical, microfluidic, shape-deformation, microwave, and various physical sensors. The use of paper in the manufacturing of various sensors opens up new possibilities both in terms of new approaches to their manufacturing and in terms of new areas of their application. However, it must be recognized that for the widespread use of paper and the appearance of paper-based sensors on the sensor market, many obstacles must be overcome.

## 1. Introduction

Paper has been with us in our lives for a long time. We use it to write letters, publish books and newspapers, and print photographs. We draw on it, use it to wrap products, and make cardboard for packaging various goods. Paper can also be an insulating material, a filter, etc. The range of its use is so wide that just a few decades ago, it was difficult to imagine any other use for paper. But economic development and environmental problems have forced us to take a new look at paper.

It turned out that the use of paper is an essential step in the transition to green technologies and circular economy [1,2,3,4,5]. The circular economy is an important element in solving climate change and other global problems such as biodiversity loss, waste, and pollution. A circular economy is a system in which materials never become waste, which helps protect the environment and restore nature faster. In a circular economy, products and materials are kept in circulation through processes such as reuse, recovery, recycling, and composting. In terms of green technologies, a key aspect of green technology is reintroduction of all devices, including electronic devices, to nature through repair or recycling methods [6]. How important this is for electronics is can be judged by the following facts. According to a United Nations report, the world generated a total of more than 62 million tons of e-waste in 2020 alone [7]. Globally, annual e-waste production is increasing by 2.6 million tons and could reach 82 million tons by 2030. It is important to note that e-waste is the fastest-growing type of waste in the world today, of which only 20% is officially recycled [8]. This is why it is very important to develop new technologies to prevent the uncontrolled growth of electronic waste. The use of paper and paper electronics is one such solution. While most polymers take hundreds of years to decompose in natural environments, cellulose paper can be decomposed within several weeks by a variety of microorganisms (fungi, bacteria, and yeasts) that exist naturally in soil [9]. What determines the prospects of paper for various applications?

Firstly, paper is made from cellulose fibers, the source of which is usually wood. Paper is mainly composed of cellulose microfibers with diameters of tens of microns and lengths up to 5 mm. This means that renewable sources are used to produce paper. Moreover, the wood used for this is far from the highest quality. Very often, waste from primary wood processing is used to make paper. That is, paper production is a rational way to use wood more fully, both from an economic and ecological point of view.

Secondly, paper is recyclable [10]. Paper products have a recycling rate of about 70%. According to the US Environmental Protection Agency’s Municipal Solid Waste (MSW) report, paper waste accounts for 27.4% of the total MSW in the United States. However, paper dominates MSW recycling at 51% [11]. The recycling rate of paper and cardboard while maintaining acceptable, although gradually deteriorating, properties of plant fibers is up to six times. Paper recycling has improved significantly in recent decades. This saves enormous amounts of energy and reduces deforestation. In addition, paper can be easily stored and transported.

Third, the unique properties of paper [12,13,14,15], such as its versatility, commercial availability, large quantity, low cost, lightweight (~10 mg/cm^2^), thin thickness, availability in a wide range of thicknesses (0.07–1 mm), high porosity, sufficient biocompatibility for bioassays, high thermal stability for robust applications, high mechanical strength for wear resistance, and elevated Young’s modulus values, make paper a promising platform for the development of flexible wearable electronics. Compared with other materials commonly used in flexible electronics such as polyethylene terephthalate (PET) and glass, paper is 200 times cheaper than PET and 1000 times cheaper than glass [13].

Paper is also well adapted to various printing technologies, which have undeniable advantages in the development of low-cost sensors and electronic devices [16,17]. Kunnari et al. [18] categorized these advantages from an eco-design (efficient use of materials) perspective. They include minimizing energy consumption at both the production and use stages, reducing the use of hazardous substances and improving recyclability. It is important to note that the advantages offered by printed electronics are unlikely to be achieved by traditional electronics manufacturing.

In addition, the rich surface chemistry enables good integration of paper with conductive materials (e.g., metallic nanomaterials, carbon nanomaterials), which are widely used in the fabrication of various devices [19]. Mass-produced paper electronics (large area printed organic electronics on paper-based substrates, “throw-away electronics”) has the potential to introduce the use of flexible wearable electronic applications in everyday life [20,21,22]. Flexible wearable electronics are the basis of many portable and disposable monitoring analytical devices for many applications, including clinical diagnostics, food quality control, safety, environmental monitoring, and healthcare, including continuous monitoring of human health in various conditions [21,23,24,25,26,27,28,29]. Moreover, since paper-based electronics are made from natural substrates of plant origin and do not contain harmful materials, they do not have a toxic effect on natural ecosystems when disposed of.

Let us now consider in more detail the direction where the advantages of paper are realized to the fullest extent, the greatest effect is achieved, and significant progress is ensured in the creation of commercially attractive devices and instruments. This direction is the development of paper-based sensors [30,31,32,33,34,35,36]. Studies have shown that paper, and especially transparent nanocellulose, is an excellent platform for the development of various optical, chemical, optoelectronic, opto-electrochemical, and electro-optomechanical sensors [24,37,38,39], where flexible, transparent, and conductive substrates or biocompatible transparent membranes capable of finely dispersing various nanomaterials and biomaterials are needed. The high porosity of paper also allows it to incorporate materials with properties important for sensor applications, but which are difficult to attach to plastic and silicon substrates. In addition, continuous pore channels that allow efficient diffusion of gases and biomolecules throughout the film matrix ensure maximum exposure of the analytes to the sensing material and thus improve the sensor signal and measurement accuracy [40].

**Figure 1:**
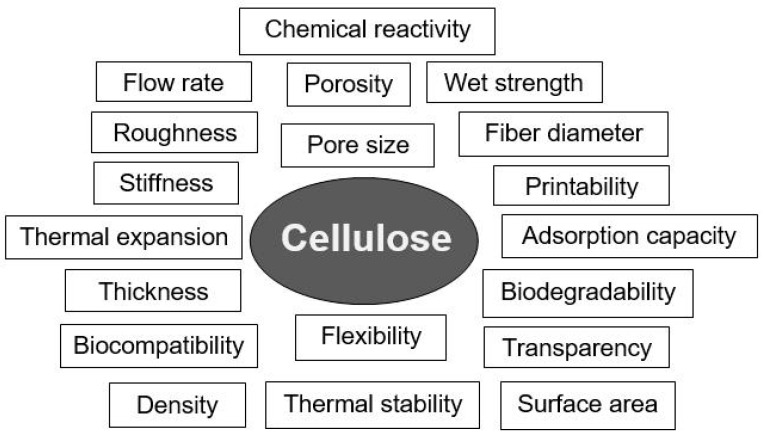
Properties of the paper that control the parameters of the paper-based sensors and electronic devices.

It is clear that each type of sensor has its own manufacturing and operating characteristics, and therefore, when developing them, various paper properties will be used to achieve optimal characteristics. Critical parameters of the paper important for practical sensor applications are shown in Figure 1. Let us now consider how these properties affect the characteristics of sensors made on their basis.

## 2. Paper-Based SERS and Plasmonic Sensors

Conventional practice for surface-enhanced Raman scattering (SERS) detection relies on adding a sample solution to an active SERS substrate or soaking the substrate in the solution and then detecting the analyte from the dried substrate by analyzing the Raman spectrum. These substrates are often made by depositing plasmonic nanoparticles (NPs) (Au, Ag) on a suitable substrate [41]. The role of the plasmonic substrate is to enhance the otherwise weak Raman signal of the analyte largely due to its localized surface plasmon resonance (LSPR) effect [42].

Experiments have shown that due to their unique properties, paper and cellulose-based materials are an important component in the development of substrates for SERS applications [43,44]. In addition, the flexibility of the paper provides excellent conformal contact with real surfaces, allowing sampling by swabs or wraps, making point-of-sample analysis convenient [45]. Paper is also an exceptional material for making the interface of surface plasmon resonance (SPR) sensors. As is known, SERS signal intensity is significantly enhanced owing to the strong electromagnetic field related to the surface plasmon resonance (SPR) of noble metallic NPs. Thus, one of the useful and key ways to improve the sensitivity of SERS and SPR sensors, as well as reduce the unwanted aggregation/agglomeration of noble metal NPs, is their synthesis/immobilization on flexible and porous substrates such as nanopaper. As is known, unwanted aggregation/agglomeration of noble metal NPs is one of the main reasons for the decrease in the sensitivity of SERS sensors. Morales-Narvaez et al. [46] and Pourreza et al. [47] were the first to report the application of nanopapers as optical sensing platforms for these applications. They utilized the advantageous features of biocellulose (BC)-based nanopaper, including its high optical transparency, functionality, flexibility, printability, and biodegradability, to develop a variety of SERS and SPR sensors based on it.

**Figure 2:**
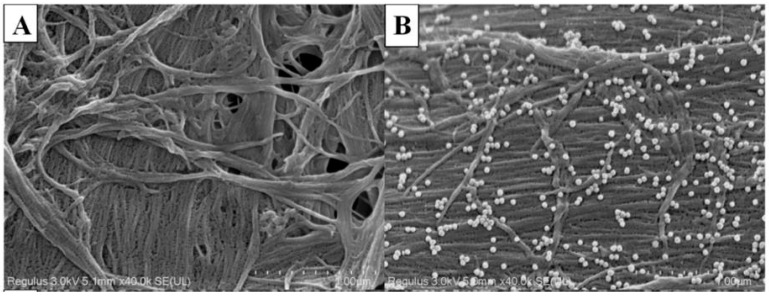
SEM images of filter paper (**A**) before Au deposition and (**B**) with Au nanoparticles adsorbed on the surface. Reprinted with permission from [50]. Copyright 2020: Elsevier.

A variety of methods can be used to deposit plasmonic NPs onto the paper substrate surface, such as dip-coating, filtration, physical vapor deposition, and printing [43]. Cellulose-based paper, in addition to minimal SERS signal interference, is capable of controlling the in situ synthesis of metal nanoparticles such as Au, Pd, Ag, and Cu, with various shapes and dimensions [48,49,50]. For example, it was shown that negatively charged celluloses, sulfuric-acid-hydrolyzed cellulose nanocrystals (CNC) [51], and 2,2,6,6-tetramethylpiperidine-1-oxyl (TEMPO)-oxidized nanofibrillated celluloses [52] are able to act as capping agents in the synthesis of spherical Ag nanoparticles. These noble metal nanoparticles generate strong surface plasmon resonance (SPR) in the visible and near-infrared regions, used in various biosensors. The three-dimensional structure of cellulose-based paper with defined porosity and morphology is an excellent platform for preventing agglomeration/aggregation of metal NPs and controlling the distance between particles (see Figure 2). Paper can also form films/membranes with defined porosity (as in the case of cellulose acetate), which is especially important in SPR analysis as it allows the control of analyte binding on multilayer immobilized antibodies with high sensitivity [53]. The initiation of capillary flow is another rationale for the use of cellulose acetate and nitrocellulose in the development of certain types of biosensors [53].

**Figure 3:**
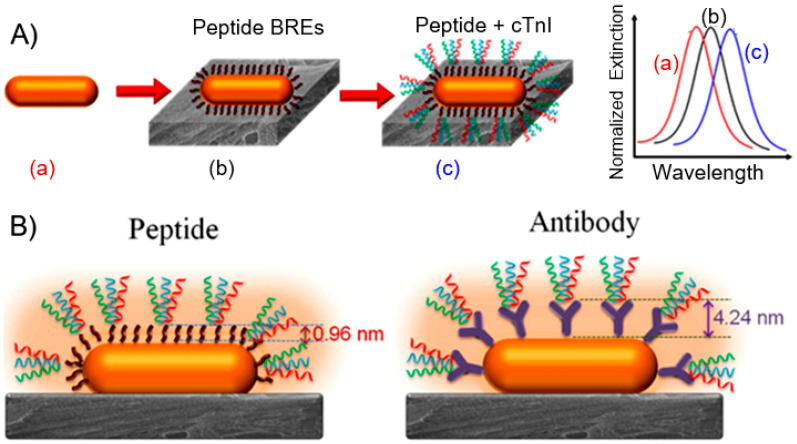
General approach used with a plasmonic paper device for the detection of biomarkers with peptide-functionalized Au NRs using their refractive index sensitivity. (**A**)—(**a**) AuNR (**b**) AuNR + peptide biorecognition elements (BREs), (**c**) AuNR + peptide BRE + Troponin I (cTnI, cardiac marker). (**B**)—Schematic showing the effect of the distance of the peptide or antibody recognition element from the surface of the nanotransducer on the refractive index sensitivity. Reproduced with permission from Ref. [58]. Copyright 2015, Nature Publishing Group.

Considering the flexibility, biocompatibility, high sensitivity, and other interesting properties of paper modified with plasmonic nanoparticles, Barajas-Carmona et al. [54] is confident that these inexpensive, lightweight, easy to manufacture and use paper-based SPR platforms will find the widest possible application. Indeed, currently, a variety of biosensors [44,47,55] and devices for the sensing of analytes in the gas phase have been developed based on nanopaper SERS and SPR optical sensing platforms. In particular, Heli et al. [56] have reported the optical monitoring of ammonia using the fabricated AgNPs/BC nanopapers. The ability to use paper as a cell culture material gives paper even more advantages in the development of biosensors, suitable for biomedical applications. For example, Tian et al. [57] developed a paper plasmonic substrate with biofunctionalized Au NPs as a highly sensitive transduction platform for rapid detection of trace bioanalytes in physiological fluids. They exploited the highly sensitive localized SPR (LSPR) wavelength shift of Au NPs due to the increase in refractive index around Au nanorods (NRs) when the bioanalyte is bound. The general concept of biosensing is schematically illustrated in Figure 3. This figure shows how the presence of a bound biomarker is observed by the spectral shift of the LSPR wavelength of Au NPs.

To highlight the comparative advantage of plasmonic paper over rigid substrates, Table 1 provides a comparison of the key performance indicators for these SERS substrates.

## 3. Fluorescent and Colorimetric Sensors

The sensitivity of fluorescent and colorimetric sensors depends largely on the interaction between the analyte and the fluorescent molecule. Compared to polymers and ceramics, cellulose has high porosity and a large surface area, which leads to improved interactions between analytes and fluorophores and chromophores. Researchers have exploited this property to detect chemicals that change the color or fluorescence intensity of fluorophores and chromophores. The work presented by Davis et al. [59] clearly shows how the properties of cellulose, its fibrillary structure, and its ability to form a three-dimensional network, as well as its processability, can be strategically exploited to improve sensor performance. For example, paper has an innate ability to adsorb and retain small amounts of liquid on its surface. At the same time, the high hydroxyl content improves the binding of chromophores and fluorophores [60,61] and ensures high yields of fluorescent groups in the cellulose matrix. All this, combined with a simple technology for immobilizing fluorophores and chromophores in the cellulose matrix, makes it possible to impart the paper properties necessary for the development of highly sensitive colorimetric and fluorescent sensors for specific applications.

For example, the anchoring of red cabbage pigment (RC) in cellulose nanofibers (CNF) [62] and carboxymethylcellulose (CMC) [63] provides sensor sensitivity to pH and NH_3_. Milindanuth and Pisitsak [64] reported that the incorporation of Rhodamine B (RhB) derivative in biocellulose (BC) nanopaper enables the development of a simple and efficient optical sensor for colorimetric determination of Cu(II) in water samples. The potential application of BC nanopaper-based platforms for colorimetric gas sensing was shown through the incorporation of KMnO_4_ within BC nanopaper. Such a platform can be applied as an optical/transparent sensor film for determining the concentration of ethylene [65].

The high content of highly reactive –OH groups present in cellulose also allows them to be functionalized, imparting a wide range of chemical variability to the surface. This can facilitate the adsorption of various molecules and improve the sensitivity and reproducibility of paper-based sensors and biosensors. Chemical functional groups introduced into the cellulose molecular chain through chemical modification can regulate and control the surface hydrophilicity, charge polarity, and specific reactivity. However, experiments have shown that oxidized or carboxylated cellulose are preferred for the immobilization of biomolecules in biosensors. This facilitates the formation of amide bonds between the amide groups of proteins and nucleic acids, as well as the carboxyl groups grafted onto cellulose molecules [66]. Such immobilization by covalent bonding is preferred because the immobilization of biomolecules by covalent bonding prevents leaching during sensor analysis. In physical adsorption, physicochemical changes in environmental conditions can disrupt the system [67].

Experiments have also shown that cellulose can be used as a scaffold for biomolecules responsible for the sensory response of paper biosensors to various analytical studies. Fluorescent sensors are particularly useful in the detection of small molecules and biomolecules, such as enzymes and DNA sequences. In particular, Golmohammadi et al. [68] used graphene oxide (GO) in combination with quantum dots (QDs), which were already coated with a specific antibody (Ab) for *E. coli* and then immobilized in BC nanopaper, for photoluminescence (PL) detection of *E. coli*. Liu et al. [69] fabricated a bioluminescent nanopaper (BLN) via a one-step immobilization of Aliivibrio fischeri (*A. fischeri*) as a bio-indicator within a BC nanopaper network. The fabricated BLN platforms were then utilized as a high-performance optical sensing platform for rapid and in situ toxicity screening of environmental contamination including polybrominated diphenyl ether (PBDE), tributyltin (TBT), and diuron. The high processability and large surface area of cellulose also leads to improved interactions between analytes, fluorophores, and biomolecules, leading to improved sensor performance [59].

It should be emphasized that in addition to its ability to immobilize chromophores and fluorophores in the cellulose matrix, cellulose can also play an active role in the recognition process. For example, the natural chirality of cellulose has been exploited in the development of a chiral fluorescent sensor for aromatic nitro compounds, such as π-basic or π-acid aromatic compounds [70], which exhibit central and axial chirality [71]. The chiral nematic phase of cellulose nanocrystals (CNCs) also allows for the implementation of chiral reflectors or polarized light detectors [72]. Such characteristics can also be used as a photonic pigment or polarization and wavelength filter by adapting mesoscopic structures [73].

The ability to easily form hydrophilic and hydrophobic zones on paper also allows the development of various assay kits for specific applications. For example, a smartphone-based assay kit, developed for point-of-care (POC) diagnosis of neonatal jaundice via PL sensing of bilirubin (BR) in infants’ blood samples, was fabricated by creating the hydrophilic test zones on the dried BC nanopaper film by an office laser printer [74].

**Figure 4:**
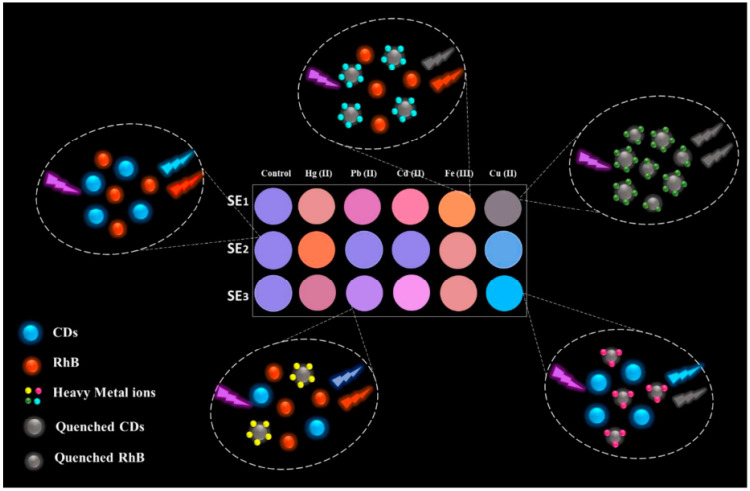
Schematic representation for discrimination of heavy metal ions using the developed nanopaper-based ratiometric fluorescent *sensor array* (NRFSA). For this purpose, the NRFSA contains three kinds of carbon dots (CDs)—Rhodamine B (RhB) nanohybrids with various capping agents (ethylendiamine (EDA), urea, glycine (Gly)) and RhB. To create the test zones with the desired patterns and hydrophobic walls, a simple and fast pattering method using an office laser printer was employed via direct printing a hydrophobic toner layer on the dried BC nanopaper films. Reprinted with permission from [75]. Copyright 2018: RSC.

The same unique physicochemical properties of nanopaper can be used to fabricate sensor arrays. Array-based sensor systems, which are also known as “chemical tongue/nose”, are when different cross-reactive sensor elements are used to provide unique fingerprint-like responses/patterns for each analyte. Sensor arrays have been proven to be an effective analytical approach for the simultaneous identification and discrimination of a wide range of analytes [75,76]. For example, Abbasi-Moayed et al. [76] have developed a BC nanopaper-based ratio metric fluorescent sensor array (NRFSA) that was employed to distinguish five heavy metal ions (Hg(II), Cd(II), Pb(II), Fe(III), and Cu(II)) through the creation of unique fluorescence fingerprint-like patterns (under UV irradiation) for each analyte (see Figure 4).

## 4. Fiber-Optic Sensors

It would seem that paper is unacceptable for developing fiber-optic sensors due to their specific configuration. But in most fiber-optic sensors, optic fiber is used only as a simple waveguide for transmitting light flux. This means that if we combine the previously discussed photoluminescent and colorimetric paper-based sensors with optic fibers, we obtain classic end-of-fiber sensors. For end-of-fiber sensors, the optical fiber acts as a conduit to carry light to and from the sample. Intensity modulation in such an optical sensor depends on the absorption or fluorescence of the analyte, indicator, or analyte–indicator complex trapped by the paper membrane located in front of the fiber. Since the probe is practically the fiber itself, a compact, highly-miniaturized sensing structure can be attained. There are other variants of fiber-optic sensors. However, they typically use either cellulose fibers [77,78] or cellulose deposited by various methods on the surface or end of glass- or plastic-based optical fibers [79,80,81,82,83]. However, this cellulose is not paper in the direct sense.

It should also be noted that end-of-fiber sensors are the simplest paper-based fiber-optic sensors. A fairly large number of such sensors have been developed to date. When using luminescent properties, the paper can be positioned directly in front of the fiber, as implemented by Eroglu et al. [84]. To fabricate H_2_S fiber-optic sensors, Eroglu et al. [84] used both the bifurcated-fiber and single-fiber configurations. The H_2_S sensing material used was filter paper pretreated with CdCl_2_. Radiation with a wavelength of 300 nm was used for excitation. The fluorescence signal was measured using a luminescence spectrometer. Studies have shown that luminescence occurs due to CdS particles formed on the surface of filter paper with a well-defined size under the influence of H_2_S [85]. The same approach was used in the development of optical fiber sensors for biochemical analysis [86,87], determination of solution pH [88,89], and detection of gases or vapors [90,91]. Typically, such sensors used cellulose membranes with the additives such as (i) Thymol blue [86], fluorescein [88], Congo red and neutral red indicator dye [92,93], and Rhodamine B (RhB) fluorescent dye [89] to determine the pH of the solution; (ii) glucose oxidase, 2,7-diaminofluorene dihydrochloride, and sodium N-(3-sulfopropyl)-3,3′,5,5′-tetramethylbenzidine [86] or carbon quantum dot (CQD)–glucose oxidase complex [87] for glucose detection; (iii) carbon quantum dots for adrenaline detection [94]; (iv) phenol red indicator dye for CO_2_ detection [91]; and (v) Nile Red dye for ethanol detection [90].

## 5. Electrical Gas and Humidity Sensors

Gas and humidity sensors are another promising area of application for paper, having important properties for these applications such as a large active surface area, an extremely high surface-to-volume ratio combined with a porous structure that provides high gas permeability, and good adsorption capacity [33,34]. Moreover, in these applications, paper can act as both a substrate for gas and humidity sensitive material [95,96] and an active material, the properties of which can be chemically functionalized in order to adapt to specific applications [97,98]. In particular, cellulose molecules contain three hydroxyl (-OH) groups that can be modified to achieve high chemical reactivity. Typically, to achieve the required sensitivity to certain gases, cellulose is functionalized with conductive materials such as CNTs, graphene, conductive polymers, or metal oxides [34,95,99]. In addition, the surface properties of the paper can also be easily manipulated by changing the printing, coating, and impregnation conditions.

The mechanism of gas and humidity paper-based sensor operation is based on the fact that cellulose fibers of paper tend to absorb moisture from the environment or interact with active gas components that are hazardous to human health [34]. The result of this interaction is a change in dielectric constant, i.e., capacity, and ionic conductivity of paper, for example, increases/decreases in resistance with decreasing/increasing concentration of gas or water vapor in the atmosphere. Based on the paper properties that change under the influence of gas and humidity, paper-based electrical gas and humidity sensors are divided into capacitive, conductometric, and impedance sensors. As a rule, regardless of the sensor type, they use interdigitated electrodes (IDE) applied to the surface of the paper substrate [100]. To improve sensitivity to certain analytes, other functional materials such as metal oxides, polymers, and carbon-based materials (CNTs and graphene) can be applied to the paper surface. Paper-based electrical sensors were considered in most detail in [33,34].

**Figure 5:**
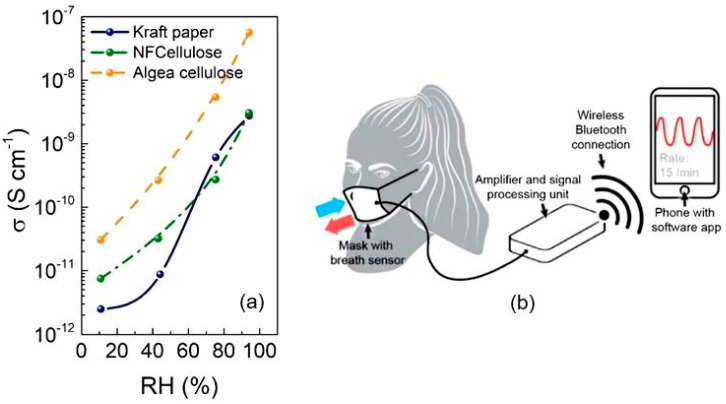
(**a**) Conductivity variation with the relative humidity (RH) for Kraft paper, wood nanofibrillated cellulose, and algae cellulose. Reprinted with permission from [103]. Copyright 2015: ACS. (**b**) Promising wearable applications of RH sensors as respiration sensors. The entire respiration monitoring system consisting of a facemask embedded with a paper respiration sensor, a signal processing circuit, and a mobile device for data display and analysis. Reprinted with permission from [98]. Copyright 2016: Wiley.

Paper-based gas sensors have been proven sensitive to hazardous gases such as ammonia, acetonitrile, toluene, cyclohexanone, and nitrogen dioxide [95,99,101]. However, from a practical point of view, the results obtained during the development of humidity sensors are more significant [34]. It turned out that with the help of such sensors it is possible to easily monitor a person’s respiratory activity, as evidenced by the ability to distinguish between different breathing patterns. Additionally, paper sensors can be integrated into a general face mask, detecting breathing status in a wearable manner and transmitting signals to the client’s mobile device (e.g., phone and tablet) to store, analyze, and display the information (Figure 5). Gas sensors and humidity sensors can also be integrated into the paper skin multisensory platform for simultaneous environmental monitoring [102]. These same sensors can also be built into diapers to signal the presence of moisture in the diapers. The use of paper ensures that such disposable sensors have a very low cost.

## 6. Electrochemical Sensors

It was found that cellulose paper can serve as an excellent platform for supporting catalysts and enzymes as it can provide both the required surface geometry and optimal hydrophilic/hydrophobic balance [104]. Thus, the fine fiber pulp and paper matrix, together with its inherent hydrophilicity, provides an excellent biocompatible microenvironment to maintain the catalytic activity of enzymes [105]. The introduction of hydrophobic functionalization of the cellulose surface allows the efficient absorption and immobilization of enzymes also through hydrophobic interactions [106]. Such properties of cellulose contribute to the successful development of high-performance electrochemical sensors, including biosensors for various applications [107,108]. Paper has many advantages for the development of biosensors, as it has the ability to retain active biomolecules for a long time.

**Figure 6:**
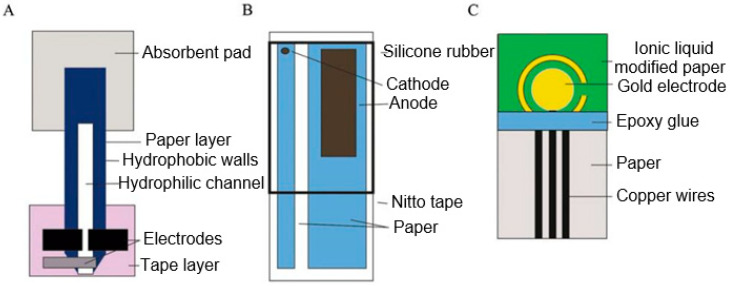
Examples of voltammetric electrochemical sensors: (**A**) stripping voltammetry measurement of Pb(II); (**B**) Clark-type oxygen electrode; (**C**) oxygen sensor based on nanoporous gold. Reprinted with permission from [13]. Copyright 2013: Springer.

Several examples of paper-based electrochemical sensors are shown in Figure 6. Constructions of electrochemical sensors shown in Figure 6 were developed by Nie et al. [109] (Figure 6A), Yang et al. [110] (Figure 6B), and Hu et al. [111] (Figure 6C). The manufacturing features and operating principles of these sensors are described in the relevant articles. It is important to note that these are very cheap sensors. For example, the price of a three-electrode device for analysis of Pb(II), developed by the Whitesides’s group [109] and having a limit of detection of 1.0 ppb, was only $0.02 per device. Various ion-selective electrodes are also adopted for the detections of Cd^2+^, Ag^+^, K^+^, NH_4_^+^, Cl^−^, and Na^+^ [112,113,114,115].

It is important to note that PB electrochemical sensors provide quick and easy ion analysis in microliters of analytes. It is also important for the operating principle of electrochemical sensors that the paper allows the transfer of ions into the *ion-selective electrode* (ISE) zone and, when wetted, ensures the closure of the electrical circuit inside the potentiometric cell. Finally, paper is a hydrophilic porous material that, through capillary forces and its network structure, can absorb and retain liquid, allowing sample processing and analyte determination to be performed in a single step. On the other hand, small volumes of absorbed analyte may be subject to relatively rapid evaporation [116].

The ability to retain metal nanoparticles or carbon-based nanomaterials, such as carbon nanotubes (CNTs) or graphene, makes nanocellulose a very promising material for the development of electrodes for various electrochemical devices [31]. Nanomaterials used to coat cellulose directly affect the specific surface area of the electrode and the immobilization of the receptor, and hence its interaction with the analyte, which directly affects the electrical conductivity and electrocatalytic properties of the electrodes and helps to increase the sensitivity threshold of sensors and improve the selectivity of their sensory response. The large surface-to-volume ratio of the paper that allows storage of reagents in large quantities, the mesoporous structure with tunable porosity in which electrolytes and ions can diffuse, and the stable electrochemical and mechanical properties of nanocellulose are an additional bonus to its use in electrochemical sensors and biosensors [117].

It is important to note that the emergence of conductive cellulose paper based on nanocomposites containing graphene [118], graphene oxide (GO) [119], single- or multi-walled carbon nanotubes (SWCNTs, MWCNTs) [120], MWCNTS [121], and nanodiamonds [122] further expands the use of cellulose in the development of electrochemical sensors [123]. Thus, due to their excellent electrochemical properties, various nanocomposites containing nanocelluloses and MWCNTs or SWCNTs have been proposed as electrode materials for amperometric detection of biomolecules. For example, a composite membrane assembled from MWCNTs and sulfated nanofibrillar cellulose (SNFC) has been used for selective and sensitive detection of various analytes in pharmaceutical products and biological fluids [121,124].

## 7. Microfluidic Analytical Devices

Another important property of paper that makes it widely used is the ability of paper to be used for the development of microfluidic devices (commonly known as microfluidic paper-based analytical devices (µPADs)), which in many cases are basic analytical instruments for human measurement and disease diagnosis [125,126,127]. The hierarchically porous structure of the cellulose paper and the abundant hydroxyl groups of cellulose fibers are advantageous for microfluidics, where liquid transportation is driven by capillary force. The hydroxyl groups together with micro-sized pores provide the capillary force that drives the transportation of solution, including biofluids, which provides the inherent capability for microfluidic devices.

**Figure 7:**
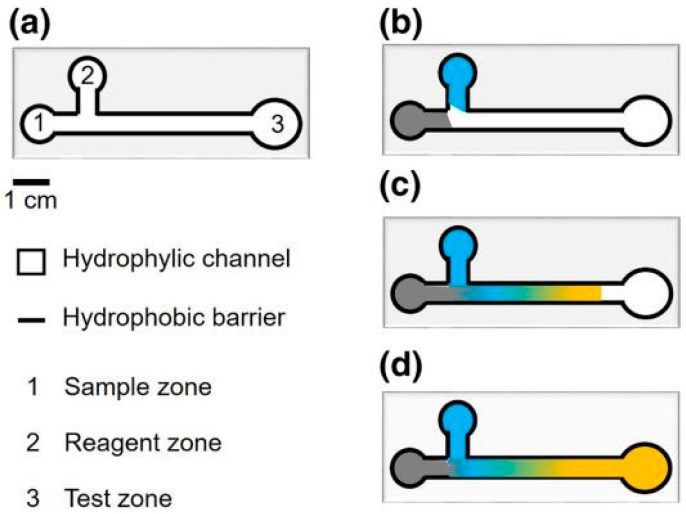
Scheme of a simple wax-based-ink-printed μPAD. (**a**) Description of the different zones of the device; (**b**) initial stage of the assay with sample and reagent confined in their zones; (**c**) sample and reagent flow through the hydrophilic channel and start to react; (**d**) end of the assay with the end-product of the reaction reaching the test zone. Reprinted with permission from [128]. Copyright 2022: Springer.

**Figure 8:**
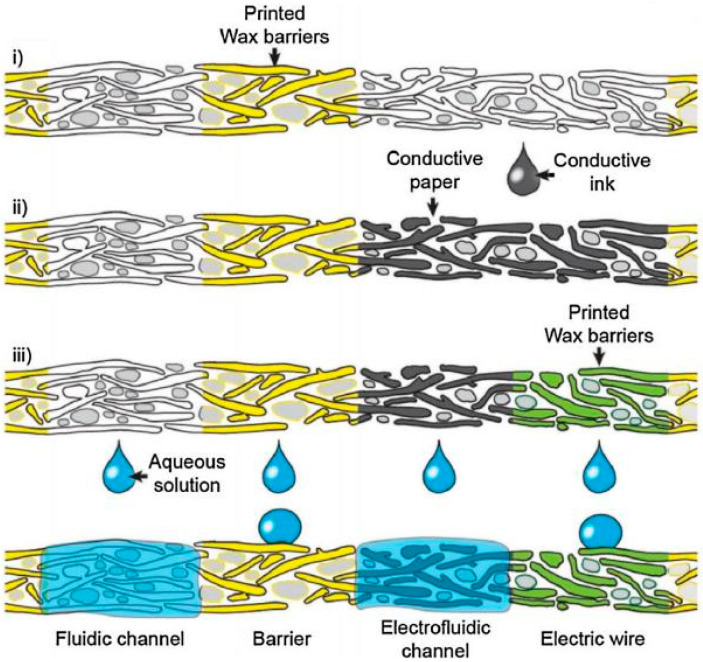
Schematic diagram illustrating the principles of fabrication of microfluidic sensors with the help of wax barriers: (**i**) Printing wax on paper to form a channel for conductive materials inside the paper. (**ii**) Adding inks to the wax-defined channels. (**iii**) Another round of wax printing to further confine the liquid microfluidic channel and fix the conductive network. Reprinted with permission from [20]. Copyright 2017: Elsevier.

One example of such µPADs is shown in Figure 7. Typically, when making µPADs, a border is applied to a paper substrate, thereby creating a physical barrier to pattern hydrophilic channels, as well as the sample and test zones (see Figure 8). Each test zone is coated with appropriate bioreceptors to detect target analytes, and microfluidic channels are responsible for the autonomous transfer of target analytes from the sample to the test zones through capillary forces [3].

Due to the natural properties of paper, liquid substances can flow through certain channels formed in the paper through capillary action without relying on external forces [123,129]. The properties of the paper, in addition to controlling the fluidic properties of the μPAD, make it possible to separate the analytes from the biological matrix [130]. At the same time, the properties of the paper matrix such as thickness, porosity, hydrophilicity, permeability, roughness, and wettability allow microfluidic behavior to be fine-tuned to meet different requirements that arise when developing sensors for various purposes [131,132,133]. In addition, thicknesses of tens and hundreds of micrometers result in a small overall volume of solutions required for analysis.

**Figure 9:**
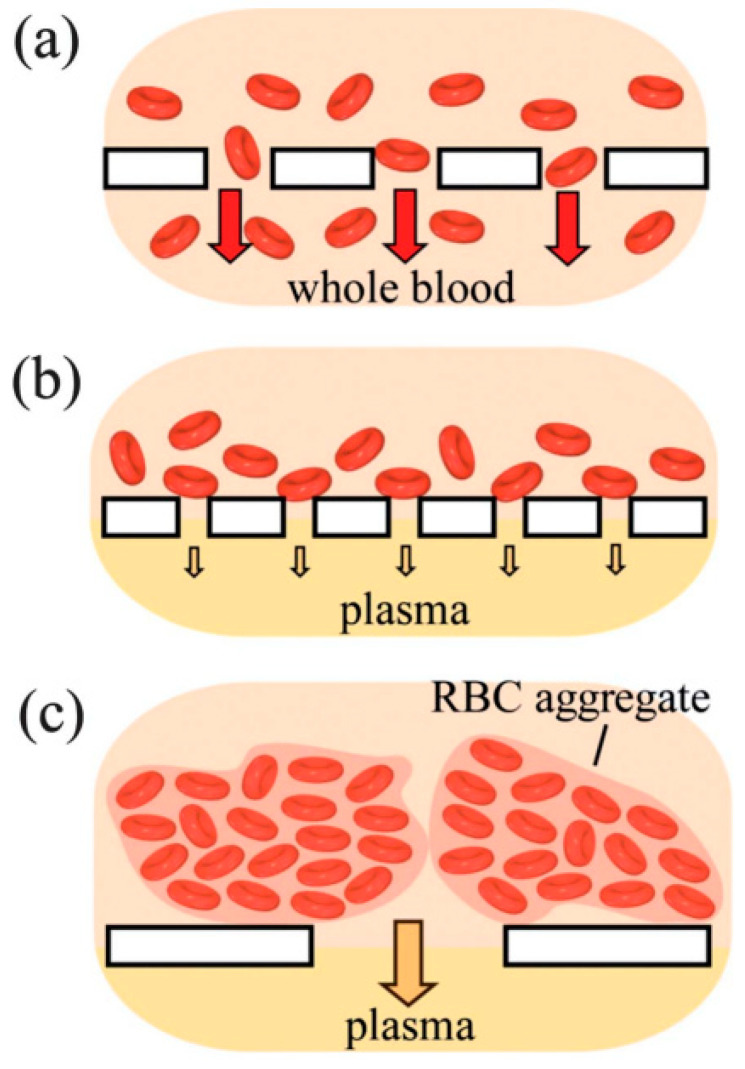
Schematic illustration of the use of red blood cell (RBC) agglutination to improve efficiency of blood plasma separation from whole blood via filtration. (**a**) RBCs can easily pass through filters with pore size as small as ~2.5 mm because of their deformability. (**b**) Filters with pores smaller than 2.5 mm block the passage of RBCs, but small pore size diminishes the flow of separated plasma severely. (**c**) Agglutinated RBCs form large multi-cellular aggregates that could be filtered out using filters with large-diameter pores, thus enabling higher rates of flow of separated plasma through the filters. Reprinted with permission from [134]. Copyright 2012: RSC.

The porous structure of paper also allows it to be used as a filter for the analysis of complex analytes. For example, Yang et al. [134] developed a simple method for separating plasma and red blood cells from whole blood using filter paper. They used Whatman #1 chromatography paper for this purpose (Figure 9). Due to blood agglutination, red blood cells could not pass through the pores of Whatman #1. However, the pores were large enough to allow plasma to pass through at a relatively high flow rate, achieving colorimetric determination of glucose concentration in a whole blood sample.

One should note that filter and chromatography papers (Whatman, Advantec, Sartorius) are most commonly used for microfluidic sampling and/or development of sensor platforms in various analytical applications [107]. Filter and chromatography papers consist of randomly laid pure wood pulp fibers and are therefore considered free of impurities [135], which are almost completely removed during the bleaching of the wood pulp to produce high-quality paper. Unlike other types of paper (e.g., office paper, cardboard), filter/chromatography papers also do not contain any structurally strengthening additives that could otherwise interfere with the performance of analytical analyses. Moreover, their relatively uniform thickness and excellent pore size result in improved adsorption and retention properties together with improved wicking characteristics [136]. However, there are no paper materials that can be considered truly free of impurities. In addition, even pure paper can sorb/desorb ions from/to the sample solution, which in turn can affect the potentiometric response of the ISE. For this reason, it is important to understand the properties of paper in terms of their impact on the potentiometric system used for various analyses.

**Figure 10:**
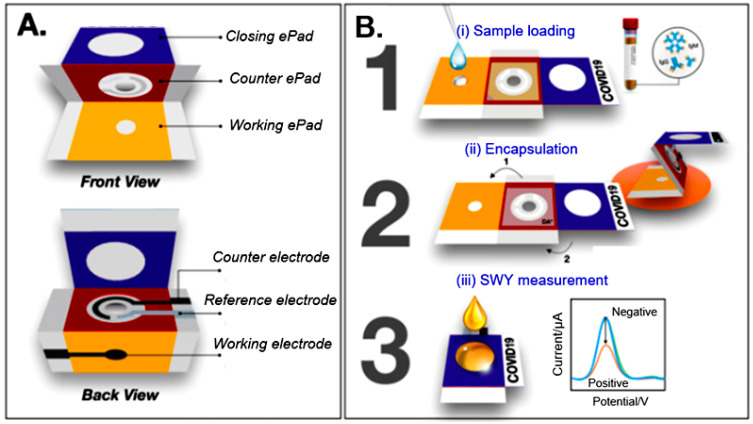
Schematic illustration of the COVID-19 electrochemical *paper*-based *analytical device* (ePAD): (**A**) device components, (**B**) detection procedure. The device was printed using a wax printer. The ePAD consists of 3 folding layers: a working ePAD, a counter ePAD, and a closing ePAD. The hydrophilic center of each zone was limited by a wax barrier, where the solution could flow through to the test zone at the bottom. Three electrodes were then screen printed at the back of the device. Reprinted from [144]. Published by Elsevier with open access.

Mechanical flexibility also allows the paper to form complex two-dimensional (2D) and three-dimensional (3D) structures [107]. Three-dimensional platforms can be composed of multiple functional layers of paper formed by folding or stacking individual layers (see Figure 10). To perform specific functions, each layer can be a combination of different paper types, with different patterns and cuts. The cuts provide a complex network of channels connected to large arrays of multiple sampling, sample processing, and detection zones. Once collected on the 3D platform, the sample percolates through the layers of the platform, rapidly dispersing and pre-processing in several steps before reaching the detection zone. Accordingly, paper-based 3D platforms are typically designed to perform multiple analyses of complex samples, such as blood, or samples that require pre-processing or maximization of footprint by performing a large number of analyses [137].

The above characteristics make paper an ideal platform for the development of microfluidic analytical devices with high sensitivity, low cost, small size, portability, ease of operation, and short analysis time [126]. The integration of such devices into wearable electronics opens up opportunities for new functions such as micro total analysis [138]. Various analytical operations typically performed in the laboratory, including sample pretreatment, transport, mixing, separation, reaction, filtration, and determination, can be easily adapted to paper-based microfluidics using smart instruments such as smartphones [139]. Leveraging the existing mobile phone infrastructure for health and environmental monitoring will speed up the diagnostic process and also help expand affordable healthcare options for existing and emerging diseases [25]. Such microfluidic devices are especially needed in kindergartens, where there is limited access to advanced analytical equipment [29,140]. The use of paper sensors will also allow low-income regions to significantly expand the range of medical services provided at a low cost. Standard medical tests performed in centralized laboratories are either unavailable in such countries or are too expensive for most citizens. At the same time, paper-based sensors, which are inexpensive and easy to use, can be used in resource-limited settings. Paper-based detection platforms also have great potential for use in remote areas and during emergency situations where fully equipped facilities and highly trained medical personnel are not available. Finally, paper/cellulose, as a widely available, lightweight, easy-to-process, and inexpensive material, allows its use as substrates for disposable sensors [3]. This technique is extensively explored for a number of analytes such as glucose, cholesterol, drugs, pH, uric acid, potassium ferricyanide, L-lactate, and alcohol in blood, urine, and sweat samples [109,141,142,143].

## 8. Mass-Sensitive Gas and Humidity Sensors

Mass-sensitive sensors include quartz-crystal microbalance (QCM) sensors, surface acoustic wave (SAW) sensors, capacitive micromachined ultrasonic transducers (CMUTs), and cantilever-based sensors. The principles of their operation are described in detail in [145,146]. The detection of gas and humidity is mainly based on the conversion of changes in mass of a sensitive material, deposited on the surface of resonator, after the absorption of gas and water molecules into a change in resonance frequency f_0_ of the active part of the sensor. This means that the sensitivity of mass-sensitive sensors largely depends on the properties of active materials, which, depending on the purpose of the sensors, must be sensitive to toxic gases, vapors of organic solvents, or humidity. These parameters primarily include the density of the sensitive material and its ability to adsorb the detected analyte. It is important to note here that paper is not typically used in these sensors. These sensors use solid-state platforms, and paper is difficult to use to functionalize the platform surface because paper cannot provide the necessary adhesion and tight contact with the platform without the use of an additional adhesive layer. In addition, due to its thickness, the paper has too much mass, which directly affects the sensitivity of the sensors. Therefore, these sensors do not use paper as the sensing element, but instead use cellulose or cellulose-based composites [30,147,148,149].

## 9. Shape Deformation Paper-Based Gas and Humidity Sensors

The use of shape deformation under the influence of moisture is one of the interesting approaches to the development of paper-based humidity sensors [150,151]. The sensing elements of these sensors normally consist of a thin moisture-sensitive film, paper in our case, which has high water-absorption properties. The key feature is that the two sides of the humidity-sensitive film have different coefficients of expansion when in contact with moisture or water vapor absorption, which leads to the bending of the film as a result of its asymmetric expansion under varying levels of humidity [152]. Indeed, typical films with symmetrical structures cannot bend due to the symmetrical changes in their volume under the influence of humidity. Therefore, the humidity sensors with the highest shape deformation have a sensing element consisting of two or more layers of materials with different water absorption characteristics [153]. However, bending is still possible, even for paper that has the same properties on both sides, if only one side is exposed to the flow of humid air. This option was considered by Wang et al. [154].

**Figure 11:**
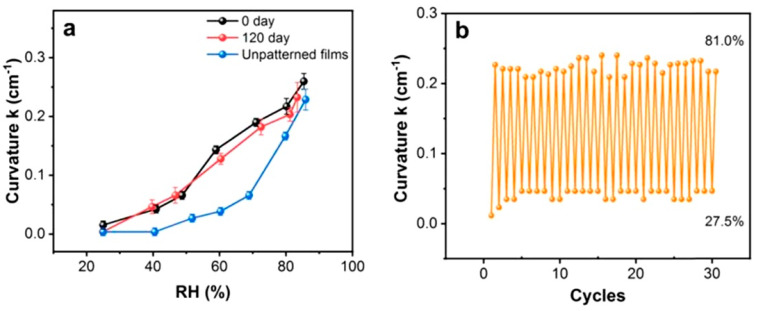
(**a**) Dependence of the curvature of patterned cellulose nanofibers (CNF)/GO films (freshly prepared and stored for 120 days), as well as unpattern ones, on humidity. (**b**) Cyclic response performances of CNF/GO film during RH switching between 27.5 and 81.0%. Reprinted with permission from [157]. Copyright 2020: ACS.

Research has shown that this simple shape-deformation-based humidity detection method can be implemented using inexpensive paper materials. Various approaches can be used for this purpose. For example, one side of a paper-based cantilever may be coated with a moisture-swellable polymer [151]. The mechanical deflection of the cantilever, caused by the swelling of the polymer, creates a tilt angle characteristic of a certain level of air humidity. One side of the paper can also be treated with cellulose stearyl ether to make it hydrophobic [155,156]. This prevents the adsorption of water vapor on that side of the paper and causes the cantilever to bend in proportion to the level of humidity. Water adsorption on the hydrophilic side expands the cellulose, while the hydrophobic side changes little, which leads to directional deformation, causing the cantilever to bend. One example of the operation of such sensors is shown in Figure 11. The curvature of the film changed from 0.012 cm^−1^ to 0.260 cm^−1^ when the relative humidity changed from 25% to 85%, and the response time was only 3–5 s.

An interesting approach to the development of a humidity sensor was proposed by Wu et al. [158]. They abandoned the use of bilayer films. In their humidity sensor, the authors took films based on cellulose nanopaper (CNP), which was derived, by using various methods, from cellulose nanofibers (CNFs). All the CNP samples were completely double-folded at room temperature and then exposed to humidity. An indicator of the moisture level was the angle between the two sides of the paper.

It is worth noting that the bending of sensors that are optimally designed is reversible and directional, and it allows cyclicity. In addition, the bending of the cantilever can be controlled by the thickness of the films applied in the sensor. In very thick films, bending is not observed. It is clear that such sensors are not very accurate. However, it is an extremely simple method that allows the monitoring of humidity without external equipment or interface electronics. Thus, with the help of these devices, it is possible to control H_2_O levels with the naked eye.

**Figure 12:**
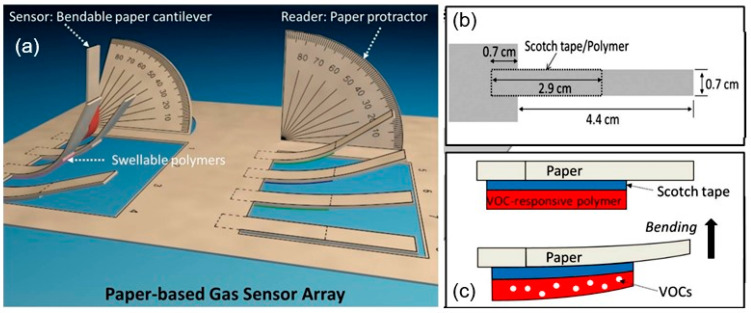
(**a**) Proposed paper-based VOC sensor array with eight different swellable paper cantilevers. The overall array dimensions were 140 mm × 160 mm. The eight-cantilever sensing arrays were split into two groups of four cantilevers, with total dimensions of 65 mm × 47 mm, while each cantilever had 44 mm × 7 mm. (**b**,**c**) Cantilever configuration without (**b**) and with (**c**) a gas-sensitive polymer layer. Polymers were spin-coated on the surface of the paper tape. Reprinted with permission from [151]. Copyright 2016: Elsevier.

The same approach can be used for detecting volatile organic compounds (VOCs) [151], as well as hydrocarbon gases such as hexane and benzene, toluene, ethylbenzene, and xylene (BTEX). VOCs and BTEX have the same effect on polymers as humidity. An example of the implementation of such sensors is shown in Figure 12. Fraiwan et al. [151] initially compared the performance of the two paper substrates: 80 µm thick A4 regular office paper and 180 µm thick Whatman^®^ chromatography paper, finding that Whatman paper resulted in better tape adhesion, the substrate was more suitable for cutting and printing manufacturing steps, and the cantilevers were more robust. Thus, the final version of the sensor array was printed onto the chromatography paper. Upon exposure to VOCs, the polymer swelling induced mechanical deflection of the cantilevers; this generated a distinctive composite pattern of deflection angles from the eight cantilevers, producing a selective fingerprint for a VOCs. This means that this device, when using various polymers, can be considered as a simple electronic nose. The various polymers possess different gas–solid partition coefficients, which will result in differences in the binding of a given vapor into a given polymer film.

The developed sensors had high stability over time, low cost, and good homogeneity. However, as we noted earlier, paper-based sensors are very sensitive to humidity, especially in field testing. Qin et al. [159] found that a 5% relative humidity difference would produce 50 times greater response than 100 ppm xylene. As discussed above, the moisture effect is mainly due to the high moisture absorption capacity of the paper substrate. Given the dramatic effects of humidity, it should be noted that eliminating false signals caused by humidity is a top priority for detecting any gases and vapors, including hydrocarbons.

## 10. Microwave Paper-Based Sensors

### 10.1. Microwave Paper-Based Humidity Sensors

Microwave sensors are another option for using the hydrophilic properties of paper with high adsorption capacity. Microwave sensors are based on the interaction between microwaves and matter. Microwave sensors utilize electromagnetic fields and devices internally operating at frequencies starting from ~300 MHz up to the terahertz range. The dispersion and dissipation of electromagnetic energy interacting with dielectric material depend upon the dimensions, shape, and relative permittivity (dielectric properties) of the material [160]. When the moisture content of the material changes, the change is reflected in the wave parameters, which can be measured. Since paper can adsorb large amounts of moisture, especially during capillary condensation, its permittivity and conductivity can vary widely, causing significant changes in the wave parameters, which is necessary when developing humidity sensors.

There are several possible ways to arrange a microwave sensor measurement. They all have different characteristics, which make them suitable for different applications [161,162,163,164]. A description of various microwave humidity sensors can be found in [145,162]. The simplest type of microwave sensors is transmission sensors. The basic geometrical configuration consists of two horn antennas, one is transmitting and one is receiving. These systems are close to their optical equivalents. In such a sensor, paper, as a sensing element, can be located between the antennas. The passage of a microwave through paper from a microwave-transmitting antenna to a microwave-receiving antenna affects both the phase and amplitude of the microwaves. Moreover, the thicker the paper, the stronger the sensor response. However, the readings of such devices are subject to the influence of external radiation sources. In addition, the approach described above is also the most dimensional.

Frequency-based or resonator-based microwave sensors and impedance sensors are another approach to the development of paper-based humidity sensors. These sensors require more complex and expensive components to operate. But these sensors are less susceptible to interference from other sources of microwave radiation. Moisture detection is made possible by shifting the resonant frequency of a resonator containing a humidity-sensitive material that absorbs the moisture. Microwave resonators can have different configurations, for example, cavity or metallic chamber, resonating when the operating wavelength exactly matches its dimensions. The introduction of a dielectric object, such as paper or cellulose, into the resonator cavity changes the resonant frequency, which correlates with the dielectric constant of the sensing layer [165]. Since the permittivity of an object depends on the moisture content, this means that the variation in air humidity, which affects the water content in a humidity-sensitive material, changes the operating frequency of the system. Thus, by measuring the change in frequency, we can determine the moisture content in a humidity-sensitive material, and therefore the humidity of the surrounding atmosphere. The output signal vs. frequency has the shape of a resonant curve, the magnitude of which decreases for increasing moisture content in the material.

**Figure 13:**
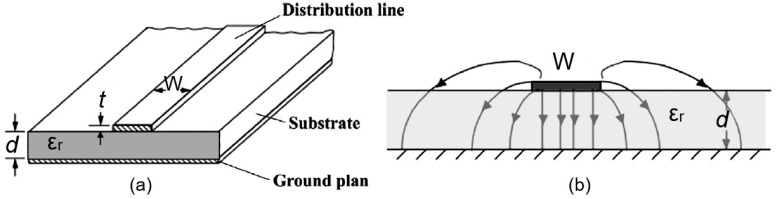
(**a**) Representation of a microstrip line. (**b**) Circulation of the electromagnetic waves between the propagation line and the ground plan (Reprinted with permission from [174]. Copyright 2015, Elsevier).

However, both transmission sensors and resonator-based microwave sensors manufactured using the conventional approach do not provide high sensitivity and small size of devices. Therefore, in recent decades, developments based on other approaches have emerged [166]. In particular, in the last decade, significant progress has been made in the development of planar microwave sensors with the microstrip patch antennas [164,167,168], slot-substrate-integrated waveguide (SIW) antenna elements [169], and coplanar waveguide (CPW) [165,170]. Such an approach is much more technologically advanced and it allows one to significantly reduce the dimensions of the device. The microstrip line [171,172] is one of the most studied propagative structures for high-frequency electronic circuits. It consists of a conductive line of distribution and a ground plan situated on both sides of a substrate (Figure 13a). The electromagnetic field orientation is represented in Figure 13b. In classic electronic circuits, the substrate is generally an insulating material. The characteristics of a microstrip line depend on its structural parameters (line width (w), length and thickness (t)) and the dielectric constant of the substrate [164]. As a rule, almost all modern microwave sensors include uniform impedance resonators (UIR) or an Electric Field Coupled Inductor Capacitor (ELC) Resonator, the resonant frequency of which is determined by its equivalent L and C components. The developed planar technology provides great opportunities for using paper, which can simultaneously be both a substrate and a sensitive element. Moreover, planar microwave sensors can be fabricated using various printing technologies [173], which are well adapted to paper. In addition, PB planar microwave sensors are compatible with many other technologies such as microfluidics, microfabrication, textiles, etc., and can be equipped with functional films, making these sensors interesting not only for humidity measurement but also for other applications such as liquid sensing, biosensors, wearable devices, physical variable measurement (e.g., temperature), etc. [164]. If we analyze the operating principles of microwave humidity sensors, we can come to the conclusion that these devices impose the same requirements on moisture-sensitive materials as capacitive sensors. Moreover, as for capacitive sensors, a decrease in the size of paper pores should be accompanied by an increase in the sensitivity of microwave humidity sensors.

**Figure 14:**
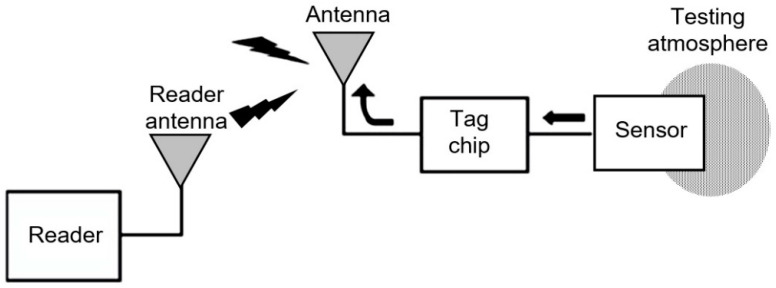
Configuration of RFID sensing systems.

At the same time, the most promising direction in the development of microwave sensors, in which paper substrates can be used with maximum efficiency, is the radio frequency identification (RFID) sensing systems, interest in which has grown dramatically in recent years [161,175,176,177,178,179,180]. Paper has a reported dielectric constant (ε_r_) of approximately 3–3.4 and a loss tangent (tan δ) of approximately 0.05–0.06 [173,181]. The relatively high loss of paper is not a critical issue for radio frequency identification (RFID) or planar structures, which have a low Q-factor, because paper is very thin. Figure 14 shows the configuration of the RFID sensing systems. RFID-empowered sensor devices have many benefits over state-of-the-art sensor components in terms of cost-effectiveness and ease of use. Typically, the structure of an RFID sensing system is comparatively simple (reader and sensor tag). It is expected that such systems will be an important element in the realization of ubiquitous environment monitoring and non-invasive control. RFID principles are also appropriate for current wireless sensor networks, which are easy to implement [169].

Virtanen et al. [182] showed that such extremely low-cost UHF RFID systems can be fabricated on flexible substrates, such as paper, using various printing technologies. It is important to note that the flexibility of RFID tags is becoming a must for virtually all applications, including wearable networks for medical systems; tracking in the pharmaceutical, agriculture, and food industries; supply chains; space; and more. In addition, the mass production of RFID tags “from reel to reel” makes paper the cheapest material for this purpose ever created [180]. Sipilä et al. [183] believe that the fabricated RFID-based humidity sensor components have a great potential to be utilized in the humidity sensing applications and also in automatic identification and supply chain control of various products, especially in the packaging and construction industry. Recently, with the rapid development of the Internet of Things and sensor technology, research on adding sensing functionality to the RFID tag becomes a hot topic [161]. Important to note that the class of sensors relying on frequency shift is most useful for remote wireless sensing because frequency shift is easier to detect than amplitude shift even in adverse signal-to-noise conditions.

### 10.2. Microwave Paper-Based Gas and Vapour Sensors

**Figure 15:**
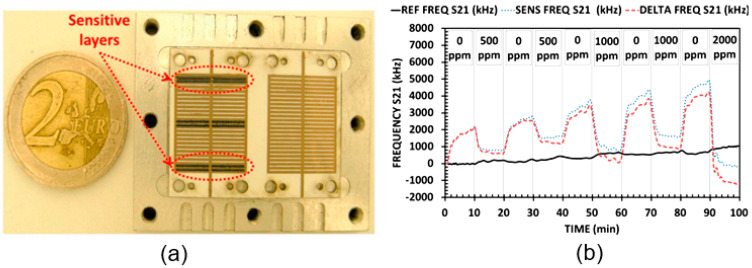
(**a**) Experimental device on paper mounted on the test cell basement. (**b**) Real-time resonance frequency variation with different ethanol vapor concentrations. The resonant frequency corresponds to the maximum loss (S_21_) of the resonator. The 0 ppm concentration corresponds to nitrogen exposure only. Reprinted with permission from [185]. Copyright 2017: Elsevier.

Experiment and simulations conducted by Bahoumina et al. (2017a, 2017b) showed that PB microwave sensors can also be used for toxic vapor detection. The sensors developed by Bahoumina et al. [184,185] were fabricated on a flexible paper substrate by inkjet printing technology. The geometry and configuration of this prototype is shown in Figure 15. The sensor platform is based on two capacitive resonators in order to provide differential detection. One resonator is functionalized with a sensitive material and constitutes the sensitive channel, while the other is considered as a reference channel and compensates the variations not related to the sensitive material. Each channel consists of two parallel networks of 50 electrodes each, spaced with a gap of 300 μm. Each resonator has two resonant modes in the frequency range up to 6 GHz.

In principle, the paper substrate itself can be used as a sensitive material, but Bahoumina et al. [184,185] decided to use a composite of poly (3,4-ethylenedioxythiophene) polystyrene sulfonate (PEDOT:PSS) and multi-walled carbon nanotubes (MWCNTs), which has proven itself in moisture detection. This choice is due to the fact that VOCs have a significantly stronger effect on polymers than on paper. In addition, due to the smaller pore diameter, capillary condensation of VOC vapors in polymers occurs at a lower concentration. This effect is very important, since, as is known, the relative permittivity of the ethanol is about 24.5 at 25 °C, compared to the permittivity of nitrogen, which is close to 1. Just as in the case of water vapor, the change in the capacitance of the sensor, leading to a change in the resonant frequency, occurs both due to the expansion of the polymer and due to a change in the dielectric constant caused by the condensation of alcohol vapor in the pores. The authors claim that their sensors were sensitive to ethanol vapor, and this sensitivity was equal to 0.9 kHz/ppm and 1.3 kHz/ppm for the sensors based on 5 and 50 sensitive layers, respectively. All detections were carried out at a fixed temperature equal to 26 °C and a humidity of 32% RH. In addition, they have shown that the sensitivity can be improved by increasing the sensitive layer thickness. Unfortunately, in real conditions, it is not possible to maintain constant humidity in the surrounding atmosphere, and therefore, with the existing high sensitivity of PB sensors to changes in humidity levels, the prospects for using PB microwave sensors for toxic vapor detection seem minimal.

There are other variants of PB microwave gas sensors. For example, Lee et al. [167] developed microwave ammonia sensors for potential application as a light-weight wireless ammonia sensor. A thin-film CNT layer was integrated with a co-planar radio frequency (RF) antenna fabricated on photographic paper substrate using ink-jet printing of silver inks. Photographic paper was chosen due to the hydrophobic nature of its coated surface. Measurements of a CNT-based sensor when exposed to low levels of ammonia reveal a resonance frequency shift of 300 MHz for a patch antenna centered around 6 GHz. It is important to note that the use of a hydrophobic substrate significantly reduces the effect of humidity on sensor readings.

## 11. Physical Sensors

Conductive paper, which has a fibrous structure, is also of interest for the development of electromechanical sensors capable of measuring mechanical stress, pressure, or displacement [19,32]. For these systems, the measurement is based on the change in capacitance or conductivity of the nanocellulose under mechanical stress. Under the influence of a tensile or compressive force, a change in the contact area between the fibers occurs, which leads to an increase or decrease in the resistance of the material, or a change in the distance between the metal electrodes applied to the paper on both sides. Nanocellulose is particularly useful due to its inherent anisotropy of properties [186,187]. This feature of nanocellulose has found application in a strain sensor capable of responding differently to forces applied parallel or perpendicular to aligned cellulose fibers [118].

**Figure 16:**
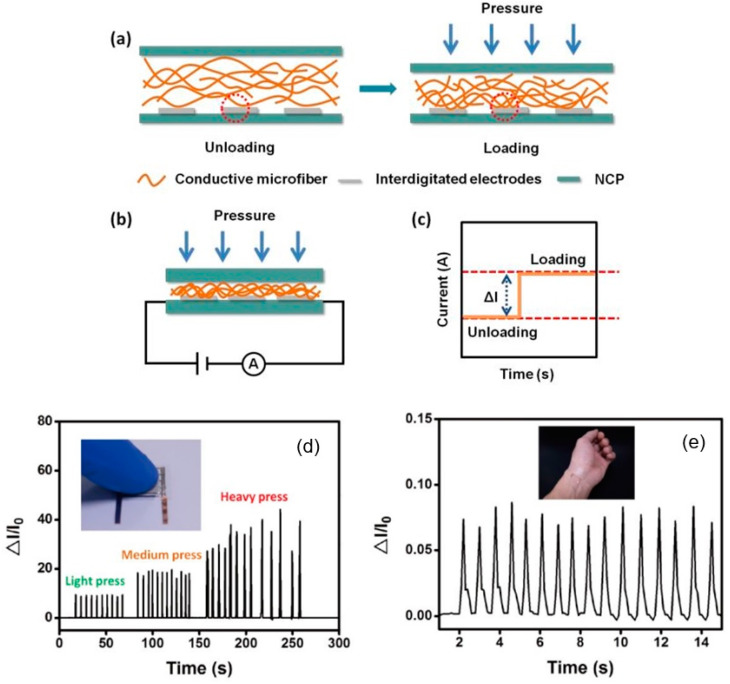
Working mechanism of all paper-based piezoresistive (APBP) pressure sensors. (**a**) Compressive deformation of a sensitive material under external pressure. (**b**) The device connection during characterizing performance of the APBP pressure sensor. (**c**) Increasing the ADBP sensor current under external pressure. (**d**) Real-time pressure sensor response. (**e**) Detection of the arterial heart pulse on the wrist, with the heart beats of 75 times per minute. Reprinted with permission from [189]. Copyright 2019: ACS.

As shown by Mun et al. [188] and Gao et al. [189], strain sensors can also be created based on thin Ag NW films deposited on the surface of flexible nanocellulose substrates. The amount of deformation in this case is estimated by measuring the change in the resistance of the surface layer of the Ag nanowire (NW) film. According to Gao et al. [189], using ordinary tissue paper or nanocellulose paper (NCP), AgNWs, and nanosilver-based conductive ink, such pressure sensors are characterized by low cost, ease of fabrication, rapid preparation for use, and easy disposal by incineration. The working mechanism of such sensors and examples of their application are shown in Figure 16. Nanocellulose substrates, unlike traditional flexible substrates [190], also promote better adhesion of the metal film. Research shows that pressure sensors can also be created based on cellulose in the hydrogel state. It was found that both compressive and tensile strains are accompanied by changes in the conductivity of the hydrogel. In [191], the observed decrease in conductivity under the influence of compressive and tensile deformations was explained by the narrowing of channels for water molecules and an increase in the resistance to ion movement.

Notably, paper-based pressure sensor designs are simple, cost-effective, scalable, and do not require complex equipment. With such devices, driving forces in different directions can be sensed, and various movements involving stretching, torsion, and bending can be controlled [19]. These sensors allow real-time and on-site monitoring of reactions to breathing, heart rate on the wrist, and even acoustic vibrations caused by external influences. Paper can also be the basis for the manufacture of temperature sensors. In particular, Nassar and Hussain [192] used the change in resistance of a silver film deposited on the surface of paper to measure temperature. The rough structure of the paper ensured good adhesion of the film and, as a result, no peeling off of the silver ink was observed. It is important to note that paper-based temperature, pressure, and stress sensors attached to a person can be easily integrated with smart wearable electronics [32,193,194]. At the same time, paper-based temperature sensors integrated with humidity sensors can be used to monitor the state of the atmosphere inside food or medicine containers [195].

The good mechanical properties of nanocellulose make it possible to create soft electronic muscle actuators based on it [191,196,197]. In particular, in [196,197], electroactive artificial muscles were created on a paper basis. Such soft electronic muscle actuators hold great promise in the era of portable consumer electronics [20] as the haptic perception capabilities they provide to users can be used in human–machine interactions, as well as in the development of reality headsets and wearable exoskeletons. Desirable actuators for these applications must conform to human motion and be biocompatible. The use of nanocellulose achieves these requirements [198,199].

## 12. Limitations and Some Approaches to Overcoming Them

As we can see, paper-based sensors are not a fantasy, but a reality. The use of paper in the manufacture of various sensors opens up new possibilities both in terms of new approaches to their manufacture and in terms of new areas of their application [200]. However, it must be recognized that for the widespread use of paper and the appearance of paper-based sensors on the sensor market, many obstacles must be overcome.

In particular, paper substrates are inferior to plastic ones in terms of mechanical strength, resistance to aggressive environments, and manufacturability. The inherent roughness and porosity of the surface makes it difficult to fabricate devices on paper, especially as their size decreases. The pronounced surface roughness of the fibrous paper structure (see Table 2) can create defects in the active layers and, as a result, deteriorate their electrical conductivity [201]. High surface roughness of the paper is especially dangerous in multi-layer devices, where one protrusion on the surface can cause a short circuit between conductors at different levels and render the device inoperable [202]. In addition, the large pore size of paper results in poor thermal performance [203].

Cellulose itself is also prone to decomposition at temperatures above 150–200 °C [204,205,206]. Excessive heating easily warps the paper and degrades the quality of the cellulose structure. The upper limit of temperatures used in the production process of paper-based devices must be below 150 °C, and this limit can only be exceeded for a very short period of time. Decomposition of cellulose leads to a decrease in the mechanical strength of paper [207]. This means that paper-based sensors and devices are low-temperature, and paper cannot be used in the high-temperature processes often used to deposit functional materials such as metal oxides. This severely limits the types of deposition processes that can be applied to paper and the amount of sensitive and active materials that can be used in paper sensors and electronic devices. In addition, the traditional paper based on organic cellulose fibers is highly flammable and easy to burn.

For paper sensors, with the exception of humidity sensors, a significant disadvantage is also the exceptional sensitivity of paper to moisture, and therefore to environmental conditions [203]. Sensors are known to lack encapsulation. This means that PB sensors have to operate in a constantly changing atmosphere. Changes in humidity and temperature have a profound effect on the fibers, the connections between them, and their size and length. It is reported that an increase in relative humidity from 25% RH to 65% RH can lead to hygroexpansion in the range of 0.1–0.4%. In addition, the intrinsic hygroscopic nature of cellulose paper inevitably compromises the stability and robustness because of unfavorable mechanical degradation and irreversible deformations in wet environments [202]. One should note that dimensional instability, which can cause cracks and separations in printed tracks, is considered one of the important problems when using fiber-based materials. In addition to changes in geometric parameters, changes in humidity lead to deterioration of the mechanical properties of paper [19] and changes in electrical conductivity and dielectric constant. The moisture content of the paper also affects the elastic modulus. High moisture content softens the material, thereby reducing the elastic modulus of the paper [208,209]. Moreover, the tendency of paper to absorb solvents results in ink permeation throughout its thickness, which reduces the lateral resolution of the printing process and causes short-circuiting of electrical connections when printing on both sides of the paper [210]. This necessitates monitoring the operating conditions of paper-based devices. Of course, the test gas can be dried before measurements. But this creates certain difficulties with the use of such sensors. In addition, due to their high sensitivity to the surrounding atmosphere, PB devices require strict storage conditions. Failure to comply with these conditions significantly reduces the shelf life and service life of PB sensors and devices [211]. It means that addressing the problem of convenient and long-term storage is crucial for promoting the widespread commercial use of paper-based sensors in the future.

Currently, the best results in the development of paper-based sensors, especially optical sensors, are achieved using transparent nanopaper substrates, fabricated using biocellolose and nanocellulose [212,213]. The use of commercial cellulose paper in optoelectronics is limited by its microscopic surface roughness, high optical haze, and low transmittance property (see Table 2). As is known, in PB optoelectronic devices, the main task is to increase the efficiency of light penetrating into and exiting from the paper substrate. Unlike microfibrillated cellulose (MFC), nanofibrillated cellulose (NFC) consists only of nano-sized structures. These fibrils have a high aspect ratio; the diameter is only 20–40 nm, while the length can be up to several microns [214]. A paper substrate consisting of nanocellulose, like regular paper, is flexible and lightweight, which meets the basic requirements for substrates for flexible electronics. Some parameters of nanopaper in comparison with regular paper and plastic are provided in Table 2. However, the synthesis method of transparent nanopaper substrates is complex and expensive [215]. Therefore, simple and efficient methods for processing nanopaper substrates, as well as simple and inexpensive methods for synthesizing nanocellolose, are very important for the future of paper-based sensors.

**Table 2:** Comparison of nanopaper, traditional paper, and plastic.

Characteristics	Nanopaper	Traditional Paper	Plastic
Surface roughness (nm)	5	5000–10,000	5
Porosity (%)	20–40	50	0
Pore size (nm)	10–50	3000	0
Optical transparency at 550 nm (%)	90	20	90
Max loading stress (MPa)	200–400	6	50
Coefficient of thermal expansion (ppm K^−1^)	12–28.5	28–40	20–100
Printability	Good	Excellent	Poor
Young’s modulus (GPa)	7.4–14	0.5	2–2.7
Bending radius (mm)	1	1	5
Renewable	High	High	Low

Source: Reprinted with permission from [216]. Copyright 2014: RSC.

**Figure 17:**
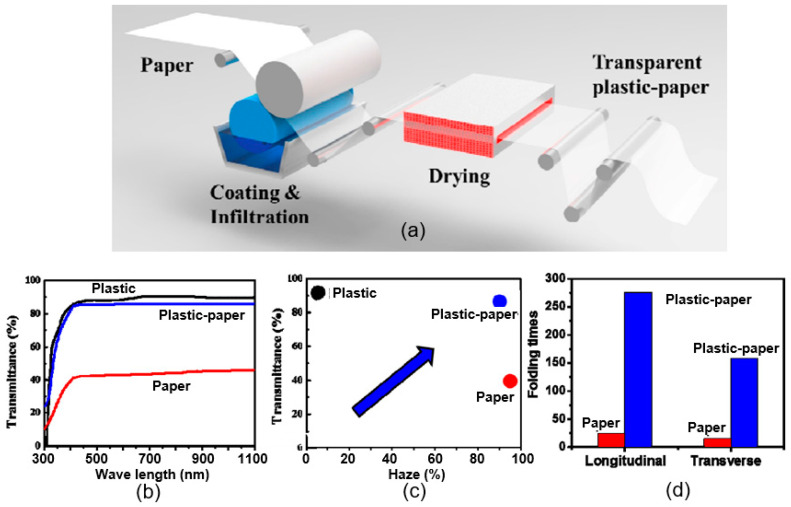
(**a**) Schematic showing roll-to-roll manufacturing of transparent plastic–paper substrates. (**b**,**c**) represent an optimized combination of high transmittance (similar to plastic) and high transmittance haze plastic–paper substrates. (**d**) Measurement of the bendability of roll paper and plastic paper in the longitudinal direction (roll paper length direction) and transverse direction (roll paper width direction). Reprinted with permission from reference [217]. Copyright 2016: RSC.

However, there is a more cost-effective method for fabricating transparent PB substrates for optical sensors [217]. It is based on a combination of paper and plastic materials (see Figure 17a). Such a plastic–paper substrate has increased optical and haze transmittances up to >85% and >90%, respectively, in a broadband wavelength (Figure 17b,c). Apart from the mechanical flexibility (Figure 17d), this new substrate is also ultra-flat and very compatible with optoelectronic device fabrication processes [217,218]. However, the presence of polymers makes it difficult to recycle such substrates.

However, more significant limitations to bringing paper-based sensors to the market are the current approaches to paper-based sensors manufacturing, which in many cases are not compatible with mass production [20]. As is known, the capability to manufacture technology at scale is essential for commercial success [29,219]. In addition, the fabrication of most flexible paper-based sensors is based on methods with which it is relatively difficult to ensure reproducibility and device stability from across batches. For example, common microfluidic materials used in academic labs, such as polydimethylsiloxane (PDMS), are not suitable for mass production [123]. While useful for prototyping and proof-of-concept studies, soft lithography is a slow fabrication process with low-throughput. Only simple but reproducible and high-performance technologies will facilitate the successful commercialization of paper-based sensors [220]. This means that in addition to cheap substrates, there is an urgent need for efficient, simple, cost-effective, industrial scalable, and green manufacturing technologies to develop more practical sensors on flexible porous substrates. Therefore, further research in this direction is urgently needed. Gong and Sinton [123] argue that the potential scalability of devices under development should be considered early in device development to ensure a streamlined transition from prototype to final product.

As can be seen from the published literature, many electronic functions, including sensing, actuation, and power supply, have been successfully implemented on paper [221]. However, it should be recognized that fully integrated paper-based sensors capable of processing and storing measurement results in accordance with the requirements of real practical applications are still in the development stage. This means that it is necessary to develop both more advanced components that make up the electronic devices and new scalable manufacturing technologies that meet the requirements of mass production. In addition, the low-resolution technologies used and the properties of paper itself limit the size of the manufactured devices, which creates difficulties in microminiaturization of these devices.

It is also worth noting that in real-world use, paper-based sensors may not be as reliable as tested in a research lab. Chemical corrosion in humid atmospheres and mechanical overload of the fragile layers on the paper surface can lead to cracking of the active layers, which can be accompanied by reduced device performance and failure, thereby further reducing the life of such paper electronics.

Of course, a number of paper properties, such as excessive roughness (Table 2) and hydrophilicity, which limit the use of paper in many applications, can be significantly improved by using special grades of paper and various methods of physical processing of paper [38,222]. For example, calendering, a method in which conventionally manufactured paper is flattened at the end of the papermaking process by passing it through stacks of hard and soft cylindrical rollers, is a well-established paper modification technique that has been shown to reduce surface roughness [223]. In particular, Vernes et al. [223] showed that surface roughness was reduced from 2–5 μm to 0.8 μm after calendering. Supercalendering provides even greater compression of the paper and hence even lower paper surface roughness. The supercalender gives paper a high-gloss finish, with the extent of supercalendering determining the extent of the gloss. Supercalendered paper is called glassine. Glassine, depending on the treatment, can have a surface roughness from 0.1 to 0.4 µm. Hyun et al. [224] showed that using supercalendering, even untreated glassine paper substrates can be used to fabricate fully in-plane printed foldable polymer-based electronic devices. Calendering also reduces the paper’s ability to absorb moisture [225].

There are also chemical methods of paper processing aimed at optimizing its properties. For example, commercial copy paper is known to be usually filled with mineral fillers such as calcium carbonate (CaCO_3_), chalk, and clays, which contribute to improving light scattering, ink absorption, and surface smoothness [202]. Removing these fillers can regulate the porosity, pore size distribution, and mechanical strength. For example, acid and/or alkali treatment of commercial paper increases the pore size and porosity from 0.31 mm and 50.3% to 12.2 mm and 82.9% [226]. There are other paper processing methods that can significantly improve its surface properties [227]. Silanization of paper results in substantially increased hydrophobicity [226]. It has been shown that laser ablation [228] can be used to improve paper surface morphology and alter surface energy, and the use of additional protective layers to seal paper devices can help reduce the impact of external influences such as air humidity on the performance of paper sensors [229]. For example, plasma polymerization can be used to create hydrophobic polymer chains on the paper surface to make it water-repellent [230].

Other interesting approaches to control paper properties include the ability to improve the hydrophobicity of paper surfaces by coating with organic or inorganic nanoparticles [231,232,233]. However, it should be kept in mind that the thickness of this protective coating often compromises the flexibility and foldability of these paper devices, making them difficult to use [234]. Without a doubt, to reduce the negative effects of encapsulation, these protective barriers must be thin; be soft; be transparent; and, most importantly, not interfere with the smooth operation of paper electronics [20]. The presence of protective polymer coatings also poses difficulties in the disposal of these devices.

In addition, it must be kept in mind that the presence of insulating coatings on the surface of the paper reduces the adhesion of the functional layers, such as metal oxides and carbon-based materials, to the surface of the paper substrate, which reduces the possibility of using such sensors in bendable devices. Therefore, it is more optimal to use hydrophobic paper as a substrate, which, while maintaining the porosity and roughness necessary for good adhesion, does not interact with water vapor. Glavan et al. [235] showed that hydrophobic paper can also be used as a substrate for electrochemical sensors. The use of hydrophobic paper in combination with nanomaterials has been found to improve the detection sensitivity of microfluidic devices [236]. As for humidity sensors, this is a more complex case, since humidity sensors can be either completely paper or sensors using additional humidity-sensitive materials. Therefore, the criteria for choosing paper as a substrate for these sensors will be different. In one case, we must proceed as described above for gas sensors, and in the other, we must use porous paper, with the size of the pores controlling the sensory response. This problem is discussed in more detail in [33,34].

As for the low thermal and electrical conductivity of paper, this problem can be solved by using thermally conductive [237,238] and electrically conductive [239,240] fillers and coatings. For these purposes, carbon-based fillers like graphene; nanotubes; and graphite, polymers, and metals, which have both high thermal conductivity and high electrical conductivity, and ceramic fillers like boron nitride, which has high thermal conductivity and is highly electrically insulating [237], can be used.

At present, high-temperature-resistant paper manufacturing technologies have also been developed [241]. For example, the addition of flame retardants can improve the fire-retardant performance of traditional paper. In this case, phosphorus-based flame retardants are usually coated on the surface of cellulose fibers, or flame-retardant components are mixed with cellulose fibers for papermaking [242,243,244,245,246]. Another strategy for the preparation of the fire-retardant paper is based on noncombustible organic fibers such as aramid fibers as the raw materials [247]. In addition, nonflammable inorganic nanofibers can be used to fabricate the fire-resistant paper [248]. But unfortunately, this fire-retardant paper does not contain cellulose, i.e., it is not paper in our understanding. Such “paper” in most cases also cannot be used in the manufacture of various types of sensors.

## 13. Summary

Of course, it should also be recognized that paper-based sensors in many cases have parameters worse than those of devices made using traditional materials and technologies [25]. For example, paper has a lower Young’s modulus (2 GPa) than silicon (130–170 GPa), resulting in a low natural resonant frequency (~25 Hz), limiting the use of low frequency or static force measurements [20]. Paper is also much less resistant than silicon-based devices to heat and atmospheric components such as water vapor, ozone, oxygen, and peroxides. Paper devices also cannot be compared to commercial devices in terms of reliability and repeatability. However, it can be assumed that the performance/cost ratio may be higher for paper-based sensors and electronics. Such sensors and instruments based on them can be targeted at low-performance applications and can significantly reduce their manufacturing costs [25].

Moreover, despite all the difficulties encountered in the development of paper-based sensors and electronics, Liu et al. [20] are confident that paper-based electronics will bring revolutionary development to the wearable electronics industry and will help improve people’s quality of life, because the use of paper and cellulose allows us to solve the problem of developing flexible and biodegradable sensors suitable for a wide range of applications, including environment monitoring, food and drug analysis, chemical warfare safety, point-of-care testing (POCT), multifunctional wearable systems such as e-skins [249], brain–machine interfaces [250], and implantable bioelectronics [251]. There is already a global market for paper-based diagnostic products valued at USD 16.39 billion in 2022 and expected to grow at a compound annual growth rate (CAGR) of 6.0% from 2023 to 2030 [252]. However, most of these paper-based diagnostics are in lateral flow and probe formats using technologies that predate the development of modern paper-based chemical and physical sensors and electrochemical paper-based analytical devices (ePADs), including electrochemical and colorimetric microfluidic devices. However, numerous challenges remain, and PB sensors are still far from reaching the sensor market [19,21,34,219,220,253,254].

It should be noted that significant progress has been made in the development and improvement of paper-based devices over the past decade. It is expected that the next decade will be even more successful, bringing breakthroughs in the accuracy and efficiency of paper sensors and biosensors, alongside the portability of wearable electronic monitoring systems, as well as solving many of the problems that limit the accuracy, consistency, and speed of real-time data collection [25]. All of this will contribute to the personalization of medicine and improvement of the primary healthcare system.

## Figures and Tables

**Table 1:** Comparison of plasmonic paper with conventional rigid substrates.

Criteria	Plasmonic Paper	Conventional Rigid Substrate
Flexibility	Yes	No
Onsite sampling	Yes	Not suitable
Additional functionalities	Yes	Not suitable
Cost	Lower	Higher
Manufacturing throughput	Depends on the fabrication method	Higher
Reproducibility	Lower-medium	Medium-higher
Transfer of liquid/solvent	Wicking of paper allows passive transport of solvent which avoids the use of pipette	Not suitable for passive transfer of liquids
Sampling	Very convenient to take sample through swabbing or as a dipstck	Not suitable for point-of-sample applications
Average enhancement factor (EF)	10^6^–10^8^	10^6^–10^8^

Source: Reprinted with permission from [43]. Copyright 2021: Elsevier.

## Data Availability

Not applicable.
